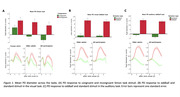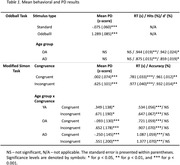# Changes in task‐evoked pupil dilation as a marker of locus coeruleus function in healthy aging and Alzheimer’s disease

**DOI:** 10.1002/alz.095455

**Published:** 2025-01-09

**Authors:** Alina Zhunussova, Clare Loane, Elif Kurt, Grazia Daniela Femminella, Sabrina Lenzoni, Millie Duckett, Martina F Callaghan, Nikolaus Weiskopf, Ray Dolan, Robert J Howard, Emrah Düzel, Dorothea Hämmerer

**Affiliations:** ^1^ University of Innsbruck, Innsbruck Austria; ^2^ Institute of Cognitive Neuroscience, University College London (UCL), London United Kingdom; ^3^ Aziz Sancar Institute of Experimental Medicine, Istanbul University, Istanbul, Istanbul Turkey; ^4^ University of Naples Federico II, Napoli Italy; ^5^ Wellcome Centre for Human Neuroimaging, University College London (UCL), Queen Square Institute of Neurology, London United Kingdom; ^6^ Institute of Neurology, University College London (UCL), London United Kingdom; ^7^ Max Planck Institute for Human Cognitive and Brain Sciences, Leipzig Germany; ^8^ Felix Bloch Institute for Solid State Physics, Faculty of Physics and Earth Sciences, Leipzig University, Leipzig Germany; ^9^ Max Planck Centre for Computational Psychiatry and Ageing, University College London, London, London United Kingdom; ^10^ Division of Psychiatry, University College London, London United Kingdom; ^11^ Center for Behavioral Brain Sciences (CBBS), Magdeburg Germany; ^12^ University Hospital Magdeburg, Magdeburg Germany; ^13^ German Center for Neurodegenerative Diseases (DZNE), Magdeburg Germany; ^14^ Institute of Cognitive Neurology and Dementia Research (IKND), Otto‐von‐Guericke University, Magdeburg Germany; ^15^ Department of Neurology, Otto‐von‐Guericke University, Magdeburg Germany; ^16^ Department of Psychiatry and Psychotherapy, Otto‐von‐Guericke University, Magdeburg Germany

## Abstract

**Background:**

Locus coeruleus (LC) is a primary source of noradrenalin in the brain and plays a complex role in human behavior. In healthy aging and Alzheimer’s disease (AD), LC cell loss has been linked to a decline in overall cognitive function. This study aimed to explore age‐ and AD‐related differences in a proxy measure of LC activity. Using pupil dilation (PD) as a non‐exclusive proxy measure of the LC‐NE system activity, we examined whether pupillometric recordings during cognitive tasks are possible in early AD and whether they reveal differences in attentional modulation in aging and AD.

**Method:**

37 subjects (14 healthy OA and 23 individuals with AD) completed an auditory and visual oddball task to assess attentional modulation; 62 subjects (22 healthy YA, 20 healthy OA, and 20 individuals with AD) completed a Simon task to assess attention and cognitive control. LC integrity was assessed using neuromelanin‐sensitive MRI.

**Result:**

A larger PD response for oddball compared to standard stimuli was observed, with no difference between OA and AD participants. In the visual task, greater PD correlated with faster reaction times (RTs) for hits in both groups, indicating the interindividual differences in PD can reflect heightened attentional involvement in aging and AD. Similarly, a consistent Simon effect, i.e., lower accuracy and longer RTs for incongruent trials, was observed in all groups, suggesting cognitive effort in discriminating between congruences. PD was higher for incongruent than congruent trials across all age groups, yet YA exhibited a less pronounced Simon effect, indicating age‐related differences in attentional resource allocation with a potentially larger need in OA and AD for attentional control on incongruent stimuli. In YA, slower RTs correlated with smaller PD in incongruent trials. YA and AD individuals with a stronger Simon effect in PD showed faster processing for incongruent trials and better performance for congruent trials, respectively.

**Conclusion:**

Using PD as a measure of attentional allocation and effort during cognitive control is possible in AD. Moreover, it allows for the assessment of interindividual differences in the extent of attentional modulation in AD. Assessing PD could be a useful tool for distinguishing between healthy aging and early AD.